# Hemostasis With Fully Covered Self-Expanding Bare Metal Stent as a Bridge to Liver Transplantation in a Patient With Acute Liver Failure and Hemodynamically Unstable Hemobilia

**DOI:** 10.1155/crgm/8873661

**Published:** 2025-08-19

**Authors:** Joshua Morny, Samuel Koebe, Michael Woods, Patrick Pfau

**Affiliations:** ^1^Department of Internal Medicine, William S. Middleton Memorial Veterans Hospital, Madison, Wisconsin, USA; ^2^Department of Radiology, University of Wisconsin Hospitals and Clinics, Madison, Wisconsin, USA; ^3^Department of Interventional Radiology, University of Wisconsin Hospitals and Clinics, Madison, Wisconsin, USA; ^4^Department of Gastroenterology, University of Wisconsin Hospitals and Clinics, Madison, Wisconsin, USA

**Keywords:** fully covered self-expanding bare metal stent, hemobilia, liver transplant, portal hypertensive biliopathy

## Abstract

We present the case of a 49-year-old man admitted for acute liver failure complicated by hemodynamically unstable hemobilia secondary to bleeding varices in the bile duct. Placement of a fully covered self-expanding bare metal stent (FCSEMS) was considered the best treatment of choice over hepatic artery embolization in this patient because of the venous source of bleeding. The success of this procedure indicates that FCSEMS can be considered as a bridge to liver transplantation in patients with acute liver failure who develop hemodynamically unstable hemobilia secondary to portal hypertensive biliopathy.

## 1. Introduction

Hemobilia is an uncommon but important etiology of upper gastrointestinal bleeding. It classically presents with jaundice, right upper quadrant abdominal pain, and upper gastrointestinal hemorrhage. The source of bleed may be arterial or venous, with the latter presenting with lower volume hemorrhage and typically self-limiting except in cases of portal hypertension where it may be in larger volume and persistent. In patients with an uncertain source of gastrointestinal bleed, recent biliary instrumentation or recent blunt, or penetrating upper abdominal trauma, hemobilia should be suspected especially when there are associated signs of biliary obstruction. Direct visualization of blood or clot emanating from the biliary tract confirms the diagnosis. The source of the bleed can also be identified with angiography [1].

## 2. Case Report

We present the case of a 49-year-old male who presented with jaundice, anasarca, abdominal pain, and melena. He was admitted for acute liver injury secondary to alcoholic hepatitis complicated by hepatorenal syndrome, portal hypertension, ascites, portal hypertensive gastropathy, and portal venous thrombosis with a Model for End-stage Liver Disease (MELD) score of 41. He was evaluated and listed for a liver transplant. This was, however, precluded by the onset of hematochezia and hypotension requiring multiple blood transfusions and dual vasopressor support.

**Figure 1:**
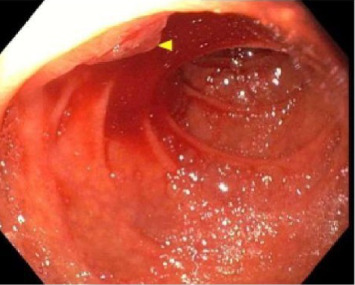
Red blood in the second portion of the duodenum with bright red blood actively hemorrhaging from the papilla with washing, visualized during initial esophagogastroduodenoscopy.

**Figure 2:**
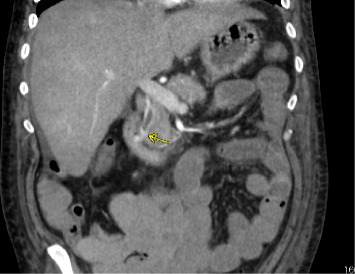
Computed tomography angiogram of the abdomen demonstrating hyperenhancement of the ampulla/papilla without active extravasation, consistent with biliary varices.

**Figure 3:**
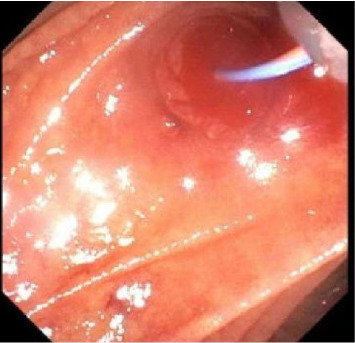
Endoscopic retrograde cholangiopancreatography (ERCP) showing cannulation of the papilla without sphincterotomy.

**Figure 4:**
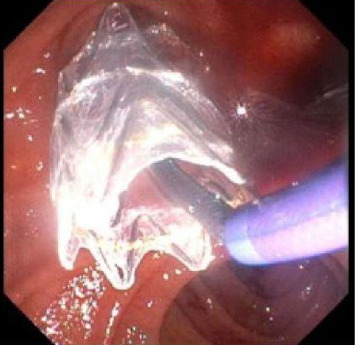
Placement of one 10 mm × 60 mm GORE VIABIL Biliary Endoprosthesis fully covered self-expanding metal stent 4 cm into the common bile duct, crossing the ampulla and papilla without sphincterotomy, during endoscopic retrograde cholangiopancreatography.

Prompted by the patients escalating hemodynamic instability with coagulopathy (Platelet: 49 and INR: 2.3) and suspicion of varices or portal hypertensive gastropathy as likely sources of bleeding, upper endoscopy was performed and demonstrated red blood in the second portion of the duodenum and at the major papilla with bright red blood actively hemorrhaging from the papilla with washing (Figure 1). In addition, there was evidence of portal hypertensive gastropathy, but no evidence of esophageal varices. Given endoscopic evidence of hemobilia, a computed tomography angiogram (CTA) of the abdomen was performed 2 hours later to ascertain the source of the bleed (arterial vs. venous). This demonstrated hyperenhancement of the ampulla/papilla without active extravasation along with small bleeding varices in the distal bile duct (Figure 2) concerning for portal hypertensive biliopathy. Given bleeding was secondary to portal hypertension, transjugular intrahepatic portosystemic shunt (TIPS) and biliary stenting were considered as options to attain hemostasis; however, the decision was made to proceed with endoscopic retrograde cholangiopancreatography (ERCP) because TIPS posed a high mortality risk in the setting of hemodynamic instability (Figure 3). Approximately 6 h after the initial upper endoscopy, one 10 mm × 60 mm GORE VIABIL Biliary Endoprosthesis fully covered self-expanding bare metal stent (FCSEMS) was placed 4 cm into the common bile duct crossing the ampulla and papilla without sphincterotomy (Figure 4). By duodenoscope visualization, hemostasis was confirmed with no further bleeding at the ampulla nor through the newly placed biliary stent. He was weaned off vasopressors and relisted for liver transplantation. Three days after ERCP, he underwent orthotopic liver transplantation with a successful duct-to-duct anastomosis. Removal of the metal stent and biliary anastomosis was completed 2 days later with no additional bleeding. Thirty days posttransplant, the patient had no further hemobilia.

## 3. Discussion

There are many possible causes of hemobilia, including iatrogenic, traumatogenic, neoplastic, inflammatory, infectious, and vascular etiologies. Rarely, hemobilia may occur in the setting of portal hypertensive biliopathy with or without prior biliary tract intervention [2]. Portal biliopathy often occurs in patients with portal vein thrombosis causing hypertension of the peri-biliary venous plexus [1]. Achieving hemostasis and maintaining bile flow is the mainstay of hemobilia treatment. Factors including, degree of hemodynamic instability, suspected source of bleeding (arterial vs. venous), and etiology of bleed determine the approach to management. Transcatheter arterial embolization (TAE) is becoming the gold standard for treating persistent and/or hemodynamically unstable hemobilia. Success rates of TAE range between 80% and 100% [3–5]. In patients with evidence of significant arterial extravasation on noninvasive imaging, TAE should be considered as the initial therapy of choice for achieving hemostasis [5]. Hemodynamically unstable patients should go directly to interventional radiology for a hepatic angiogram and embolization or to surgery. Endoscopic biliary stent placement is generally considered the initial therapy of choice for hemodynamically stable hemobilia without clear evidence of arterial bleeding [2]. Hemobilia in this setting may not respond to treatment by interventional radiology with arterial embolization because an arterial source is not the cause of the bleeding but rather due to varices in the bile duct [6]. Both plastic and metal stents have been shown to achieve immediate hemostasis by creating a tamponade effect and maintaining duct patency. FCSEMS have replaced plastic stents because they have a superior tamponade effect with a larger diameter and significantly more radial force [7].

A few case reports have demonstrated hemostatic success after placing FCSEMS in patients with hemodynamically stable hemobilia in the setting of portal biliopathy [7–9]; however, there have been no case reports outlining use of FCSEMS as a bridge to liver transplant in a patient with hemodynamically unstable hemobilia in the setting of portal biliopathy and acute liver failure. Services for both ERCP and angiographic embolization are available 24 h at our institution and can be mobilized and performed within 1–3 h in emergent cases. ERCP with FCSEMS was not performed at the time of initial upper endoscopy because the endoscopist was not credentialed to perform ERCP. ERCP is generally avoided in critically ill patients who are inadequately resuscitated with ongoing hemodynamic instability; nonetheless, ERCP with biliary stent placement was considered the best treatment of choice over hepatic artery embolization in our critically ill patient because of the suspected venous source of bleeding. Hepatic artery embolization would have been therapeutically ineffective in our patient due to the venous source of bleeding. Moreover, given the presence of a thrombosed portal vein, embolization could have led to significant hepatic ischemia affecting both the native and transplanted liver, as hepatic perfusion relies on both the hepatic artery and portal vein. This would have further worsened his already critical condition. TIPS placement was also considered, but due to concerns about high mortality risk in the setting of hemodynamic instability, this option was deferred. Sphincterotomy was not performed in our patient due to increased bleeding associated with coagulopathy and the risk of cutting into the varices. Our patients' bleeding stopped at the time of stenting, allowing him to stabilize clinically and undergo transplantation. Transplantation decompressed the portal hypertension as demonstrated by the ability to remove the FCSEMS 2 days posttransplant with no bleeding and no evidence of portal biliopathy. Whereas biliary stenting for portal biliopathy was performed in purely palliative patients according to previous case reports, and its success in our patient indicates that bare metal stenting may be considered as a bridge to liver transplantation in patients with acute liver failure complicated by hemodynamically unstable hemobilia secondary to portal biliopathy.

## Data Availability

The data that support the findings of this study are available from the corresponding author upon reasonable request.
